# Increases of peripheral blood pressure in men with cervical spinal cord injuries

**DOI:** 10.1097/MD.0000000000041887

**Published:** 2025-03-21

**Authors:** Ken Kouda, Takeshi Nakamura, Yoshi-ichiro Kamijo, Motohiko Banno, Yumi Koike, Tomoyuki Ito, Fumihiro Tajima

**Affiliations:** a Department of Rehabilitation Medicine, Wakayama Medical University School of Medicine, Wakayama, Wakayama, Japan; b Department of Rehabilitation Medicine, Yokohama City University Graduate School of Medicine, Yokohama, Kanagawa, Japan; c Department of Rehabilitation Medicine, Dokkyo Medical University Saitama Medical Center, Koshigaya, Saitama, Japan; d Department of Health Science, Aichi Gakuin University, Nisshin, Aichi, Japan; e Chuzan Hospital, Okinawa, Okinawa, Japan.

**Keywords:** capillary blood pressure, cervical cord injuries, pressure injury, sympathetic nerve

## Abstract

Patients with cervical spinal cord injuries (CSCI) experience hypotension mainly caused by attenuated total peripheral resistance. Some physicians and nurses believe that this peripheral blood pressure (PBP) decrease in patients with CSCI results in the formation of pressure injuries. We hypothesized that the measurement of PBP, such as arteriole and capillary pressure, could be a simple and efficient means of detecting skin and deep tissue damage in patients with CSCI. However, PBP in the skin has not been measured in patients with CSCI. The purpose of this study was to investigate PBP in patients with CSCI. Eleven men patients with CSCI with a lesion between C6 and C7 (American Spinal Injury Association score A, average age 32.5 years) and 13 healthy participants (average age 40.1 years) participated in the study. To measure PBP, laser Doppler blood flowmetry with a fixed pressure transducer was gently applied to the pretibial skin, and the pressure when skin blood velocity was zero was determined as the PBP. The mean blood pressure (MBP) was measured simultaneously. There was a significant correlation between MBP and PBP in patients with CSCI, but not in healthy individuals. A significantly lower MBP was observed in patients with CSCI (means ± SE; 76.7 ± 3.9 mm Hg) than in healthy individuals (means ± SE; 91.0 ± 4.0 mm Hg). PBP in patients with CSCI (means ± SE; 55.7 ± 2.0 mm Hg) was significantly greater than in healthy individuals (means ± SE; 45.9 ± 2.3 mm Hg). Transection of the sympathetic nervous system from the medulla to the peripheral nerves in patients with CSCI results in decreased MBP and increased PBP. We suggest that cervical spine transection diminishes the tonic impulses of sympathetic nerves to resistant vessels in patients with CSCI.

## 1. Introduction

In patients with cervical spinal cord injury (CSCI), efferent sympathetic nerve activity from the cerebral or medullary cardiovascular regulating components to the heart and peripheral vessels is blocked.^[1,2]^ Therefore, patients with CSCI have hypotension mainly caused by attenuated total peripheral resistance. In addition, most physicians believe that capillary pressure in patients with CSCI could be attenuated and easily form pressure wounds.^[3]^ Pressure wounds are recognized as a major health issue due to the loss of mobility, lack of sensation, and impaired microvascular function in patients with CSCI. In addition, pressure wounds are ischemic, resulting from pressure and shear forces that occlude cutaneous and subcutaneous blood flow.^[4]^ Although techniques for assessing skin blood flow have progressed, the methods of measuring peripheral pressure are limited. We hypothesized that the measurement of peripheral pressure, such as arteriolar and capillary pressures, could be a simple and efficient means of predicting lasting tissue damage in CSCI.

In previous studies,^[5,6]^ capillary pressure has been measured in the capillaries of exposed animal tissues and in large capillary loops at the base of the fingernail in humans using the micropipette method by direct cannulation of the capillaries. Shore et al demonstrated that capillary pressure can be estimated using a unique noninvasive method.^[7,8]^ Furthermore, a study on skin perfusion using laser Doppler perfusion imaging has been conducted in patients with spinal cord injuries in the past decade.^[9]^ However, blood pressure (BP) in capillaries has not been measured in patients with CSCI. This study aimed to investigate capillary BP in able-bodied controls and individuals with CSCI. We assumed that laser Doppler perfusion imaging might include other peripheral blood flow than capillary blood flow, therefore we use the terms “peripheral blood flow” and/or “peripheral blood pressure” in this paper.

## 2. Materials and methods

### 2.1. To measure peripheral blood pressure

**Figure 1.**
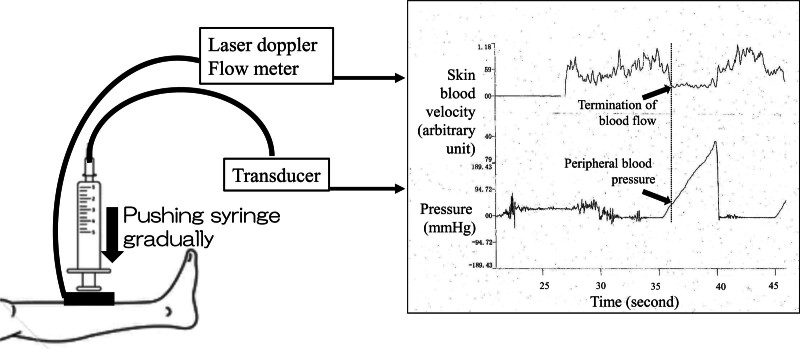
Illustration of the system and sample data used to measure peripheral blood pressure. The top of the syringe was connected to a transducer through a tube to measure the pressure inside the syringe. A laser Doppler flowmetry probe was attached to the piston of a glass syringe. The pressure inside the syringe was adjusted to zero before pushing the pretibial skin, and the piston was gently and slowly pushed towards the pretibial skin of the participant. The skin blood velocity was measured simultaneously using laser Doppler flowmetry and pressure on the pretibial skin. When the skin blood velocity was constant, the pressure on the pretibial skin is detected as peripheral blood pressure.

Figure 1 shows a schematic diagram of the device. Two systems, laser Doppler flowmetry (OMEGAFLO; OMEGAWAVE, Tokyo, Japan) and a pressure transducer (Edwards Lifesciences, Tokyo, Japan), were integrated. It consists of a laser Doppler flowmetry probe attached to the piston of a glass syringe and a transducer for pressure measurements inside the syringe. The top of the glass syringe was connected to a pressure transducer via a tube (Edwards Life Sciences). The pressure inside the syringe-adjusted zero calibration indicated the pressure on the pretibial skin. The skin blood velocity was measured simultaneously using laser Doppler flowmetry. When the skin blood velocity was constant, the pressure on the pretibial skin was determined as the peripheral blood pressure (PBP).

### 2.2. Subjects

Eleven men patients with CSCI with a lesion between C6 and C7 (American Spinal Injury Association score A, average age ± SE, 32.5 ± 2.8 years) and 13 healthy participants (average age ± SE, 40.1 ± 3.5 years) were included. The individual baseline characteristics of the patients with CSCI are shown in Table 1, and the baseline characteristics of both groups are presented in Table 2. There were no significant differences in age, height, or pulse rate between the 2 groups. Body weight, systolic blood pressure (SBP), diastolic blood pressure (DBP), and mean blood pressure (MBP) were significantly lower in the patients with CSCI than those in the control participants (*P* < .05).

**Table 1:** Characteristics of the CSCI subjects.

Subject	Age, yr	Level of spinal lesion	Time since injury, yr	Present pressure ulcers	Past history of pressure ulcers
1	54	C6	5	−	+
2	21	C6	4	−	+
3	37	C6	34	−	−
4	28	C6	8	−	+
5	32	C6	10	−	+
6	30	C6	12	−	+
7	24	C7	7	−	+
8	38	C7	18	−	+
9	33	C6	11	−	+
10	20	C6	2	−	+
11	41	C7	1	−	−

CSCI = cervical spinal cord injuries, C = cervical, + = present, − = absent.

**Table 2:** Baseline characteristics.

	CSCI subjects (n = 11)	healthy subjects (n = 13)	*P*
Age (yr)	32.5 ± 2.8	40.1 ± 3.5	.131
Body weight (kg)	56.7 ± 2.3	64.7 ± 2.2	.028
Height (cm)	169.1 ± 1.7	171.2 ± 1.5	.382
SBP (mm Hg)	108.6 ± 3.9	120.8 ± 4.0	.049
DBP (mm Hg)	60.7 ± 4.0	76.1±4.4	.023
MBP (mm Hg)	76.7 ± 3.9	91.0 ± 4.0	.023
PR (beats/min)	62.7 ± 3.2	71.8 ± 3.0	.069
Capillary blood velocity (kHz)	0.49 ± 0.0	0.52 ± 0.0	.57
Blood flow (mL/min/100 g)	9.0 ± 0.2	9.7 ± 0.1	.001

Values are means ± SE.

DBP = diastolic blood pressure, MBP = mean blood pressure, PR = pulse rate, SBP = systolic blood pressure.

The study protocol conformed to the Helsinki Declaration and was approved by the Human Research Ethics Committee of Wakayama Medical University School of Medicine. Written informed consent was obtained from all the participants.

### 2.3. Study protocol

Measurements were performed in the afternoon with the participants in the supine position with their legs at heart level. All tests were started in a quiet temperature-controlled room (26 ± 1°C). The experimental procedures began after a resting period of at least 30 minutes. The participants refrained from smoking and caffeine-containing drinks the day before the measurements.

### 2.4. Measurements

The pressure inside the syringe was adjusted to 0 mm Hg. To measure the PBP, the probe was gently applied to the pretibial skin of the participant and compressed gradually and continually for 5 seconds until the output signal of the flowmetry showed constant values. This measurement was performed thrice for each subject by the same experienced operator. Data were collected using a data acquisition system (Bimutus II system; KISSEI COMTEC, Nagano, Japan) and simultaneously displayed and recorded in a spreadsheet format on a personal computer.

Simultaneously, SBP and DBP were non-invasively measured by the auscultatory method using a sphygmomanometer on the contralateral arm of the blood flow-measuring leg. MBP was calculated using the formula MBP = (SBP − DBP)/ 3 + DBP.

### 2.5. Statistical analysis

Data were expressed as mean ± SE. Capillary BP and MBP were compared between men with CSCI and healthy patients by using a paired Student *t* test. Pearson’s correlation analysis was performed to evaluate the relationship between PBP and MBP. Statistical analyses were performed using IBM SPSS (version 20.0; IBM Corp., Armonk, NY). All statistical tests were 2-sided and were considered statistically significant at *P* < .05.

## 3. Results

Skin blood velocity did not differ between the 2 groups. Blood flow in patients with CSCI (mean, 9.0 ± 0.2 mL/minute/100 g; range, 8.2–9.7 mL/minute/100 g) was significantly lower than that in the healthy participants (mean, 9.7 ± 0.1 mL/minute/100 g; range, 8.9–10.3 mL/minute/100 g; Table 2).

**Figure 2.**
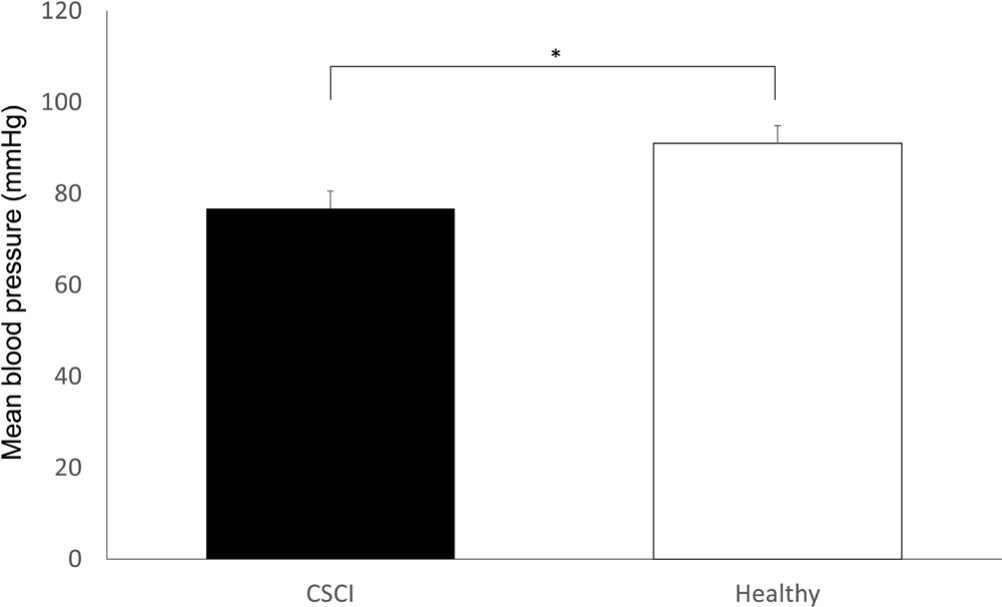
Mean blood pressure (MBP) of the arm during measurement of the contralateral pretibial peripheral blood pressure measuring. MBP in patients with cervical spinal cord injury is significantly lower than healthy individuals (healthy). *P < .05.

**Figure 3.**
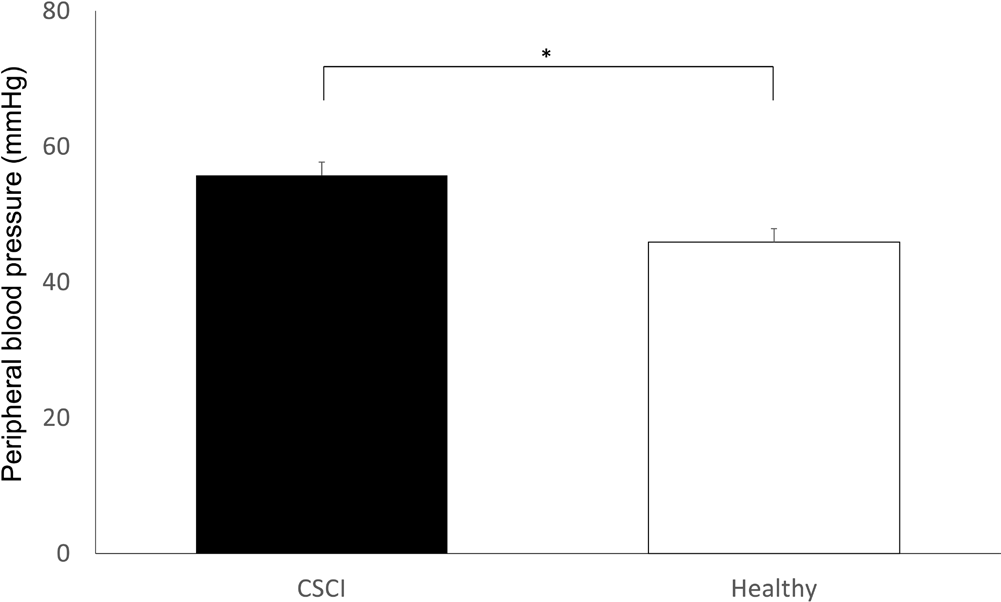
Peripheral blood pressure (PBP) of the pretibial skin during measurements of the ipsilateral arm mean blood pressure. PBP in patients with cervical spinal cord injury is significantly higher than healthy individuals (healthy). *P < .05.

A significantly lower MBP was observed in patients with CSCI (mean, 76.7 ± 3.9 mm Hg; range, 55.7–93.3 mm Hg) than in healthy participants (mean, 91.0 ± 4.0 mm Hg; range, 72.7–121.7 mm Hg, *P* < .05; Fig. 2). PBP in patients with CSCI (mean, 55.7 ± 2.0 mm Hg; range, 45.1–69.4 mm Hg) was significantly greater than in control participants (mean, 45.9 ± 2.3 mm Hg; range, 35.3–65.3 mm Hg, *P* < .01; Fig. 3).

**Figure 4.**
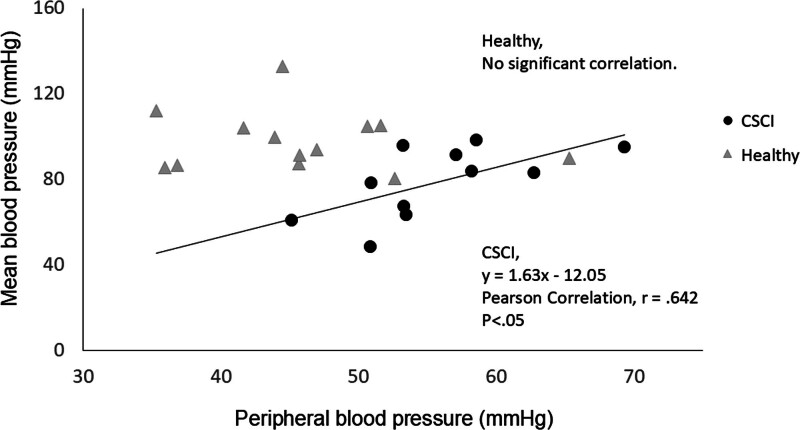
Pearson correlation between mean blood pressure (mm Hg) and peripheral blood pressure (mm Hg) in patients with cervical spinal cord injury (CSCI) and healthy individuals (healthy). The solid line represents the linear correlation. Pearson correlation, *r* = 0.642 (*P* < .05). There is no significant correlation in healthy. CSCI = cervical spinal cord injury.

There was a positive relationship between MBP and PBP in patients with CSCI (Pearson correlation: *r* = 0.642, *P* < .05); the scatter plot and linear regression results for this relationship are presented in Figure 4. In contrast, PBP did not significantly correlate with MBP in healthy participants.

## 4. Discussion

The major finding of the present study was that PBP was significantly greater in patients with CSCI than in control participants, regardless of the MBP results. Cardiovascular control in individuals with large spinal cord lesions (quadriplegia) dissociates from any cerebral or medullary regulatory components. Pressure wounds are a major and severe complication of spinal cord injury. Prevention of pressure wounds is preferable to treatment. Frequent change of position prevents tissue injury due to pressure, the present results supports the physiological background of the clinical traditional nursing method. Patients with CSCI should be advised to shift their weight every 2 hours.^[10]^ Before the present experiment, we hypothesized that PBP in patients with CSCI would be lower than that in able-bodied controls because their MBP should be much lower than that in healthy individuals. However, hypotension in patients with CSCI should be caused by a decrease in arteriole resistance, and MPB should be close to PBP, which means that MPB decreases and PBP increases in patients with CSCI. These pathophysiological aspects demonstrate the present results of increased PBP.

The present results show a significant Pearson correlation between MBP and PBP in patients with CSCI but not in healthy individuals. We suggest that diminished sympathetic signals from the central nervous system to resistant vessels in patients with CSCI induce direct reflection from the MBP to the PBP, but the existence of sympathetic signals in healthy individuals could interrupt direct reflection.

It is well-known that aging affects arterial blood pressure, and it may also impact peripheral blood pressure. In this study, although there was no significant age difference between the healthy individuals and the patients with CSCI, the healthy individuals tended to be older. Therefore, we cannot completely rule out the impact of aging on peripheral blood pressure. Previous reports have indicated that peripheral blood flow decreases with age.^[11]^ However, in our study, peripheral blood flow was significantly higher in older healthy adults. Based on these results, we believe that aging did not strongly affect peripheral blood flow or peripheral blood pressure in this study. Additionally, the body weight of the healthy individuals was significantly higher than that of the patients with CSCI. To date, no studies have demonstrated a link between body weight and peripheral blood flow or pressure. While some research has examined the relationship between peripheral arterial disease (PAD), caused by atherosclerosis of the peripheral arteries, and body weight, most findings show no correlation between PAD onset and body weight.^[12–14]^ Although we cannot entirely dismiss the possibility that body weight might influence peripheral blood pressure, we assume its effect is minimal.

With this alternative setup, the capillary pressure can be measured quite well and within a relatively limited time. The capillaries of the fingernail fold were visualized by means of a capillary microscope with motor focusing in combination with a video circuitry as described previously.^[15]^ The assessment of cutaneous microcirculation by laser-Doppler flowmetry has been used in humans to evaluate cutaneous blood flow and provides noninvasive, real measurements of local tissue blood flow.^[16]^ On the other hand, laser-Doppler flowmetry is not a true measure of microcirculatory blood flow, but capillary blood flux. Laser-Doppler flowmetry has been demonstrated to be an accurate alternative to other accepted and more complex techniques of flow measurement, such as radioactive xenon, fluorescein appearance, skin temperature, transcutaneous carbon dioxide tension, and plethysmography.^[17]^ The present findings have clinically great meanings. To date, there have been no suggested pathophysiological benefits of sympathetic nervous system dysfunction in patients with CSCI. However, if patients with CSCI are carefully repositioned in the hospital, a slightly higher peripheral blood flow should be maintained, and pressure wounds can be prevented.

There are several limitations to this study: we recruited only 11 participants with cervical spinal cord injuries, all of whom had an American Spinal Injury Association score of A, resulting in a small sample size; we did not collect detailed information on the participant’s smoking, drinking, and exercise habits, which could influence peripheral blood pressure; and there were no direct measurements of peripheral blood pressure. Further research is needed to directly measure peripheral blood flow.

## 5. Conclusion

The present study demonstrated that transection of the sympathetic nervous system from the central nervous system to the peripheral nerves in patients with CSCI resulted in an increase in peripheral blood pressure. We suggest that cervical spine transection diminishes the tonic impulses of sympathetic nerves to resistant vessels in patients with CSCI. When we consider good sheeting and bed position in patients with CSCI, the possibility of pressure wound formation is decreased compared with that in persons with lower spinal cord injuries.

## Acknowledgments

This study was supported by Nachi Katsuura City Foundation for Research.

## Author contributions

**Conceptualization:** Ken Kouda, Takeshi Nakamura, Fumihiro Tajima.

**Data curation:** Ken Kouda, Takeshi Nakamura.

**Formal analysis:** Ken Kouda, Yoshi-ichiro Kamijo.

**Funding acquisition:** Fumihiro Tajima.

**Investigation:** Ken Kouda, Takeshi Nakamura, Yoshi-ichiro Kamijo, Motohiko Banno, Yumi Koike, Tomoyuki Ito, Fumihiro Tajima.

**Methodology:** Ken Kouda, Fumihiro Tajima.

**Project administration:** Ken Kouda, Fumihiro Tajima.

**Validation:** Fumihiro Tajima.

**Visualization:** Ken Kouda.

**Supervision:** Takeshi Nakamura, Yoshi-ichiro Kamijo, Fumihiro Tajima.

**Writing – original draft:** Ken Kouda.

**Writing – review & editing:** Takeshi Nakamura, Fumihiro Tajima.
